# Retraction: Water hyacinth: a possible alternative rate retarding natural polymer used in sustained release tablet design

**DOI:** 10.3389/fphar.2016.00141

**Published:** 2016-06-01

**Authors:** Frontiers in Pharmacology Editorial Office

The journal retracts the 11 June 2014 article cited above. Based on information discovered after publication and reported to Frontiers in November 2015, the article was examined, revealing that the complaint was valid and that the article should be retracted due to insufficient scientific quality. The retraction of the article was approved by the Field Chief Editor of *Frontiers in Pharmacology*. The authors do not agree to the retraction or to the notice.

